# Undesirable Weight Change and Reduced Sleep Quality in University Older Employees During the COVID-19 Pandemic

**DOI:** 10.1093/geroni/igab046.248

**Published:** 2021-12-17

**Authors:** Hamza Butt, Kathie Insel, Kendra Jason, Mark Wager, Dagoberto Robles, Yunjia Yang

**Affiliations:** 1 University of Arizona, Tucson, Arizona, United States; 2 Behavioral and Social Science, Tucson, Arizona, United States; 3 UNC Charlotte, Charlotte, North Carolina, United States

## Abstract

This study investigated the occurrence of undesirable weight change (UDWC) and reduced sleep quality (RSQ), and major factors associated with these changes during COVID-19 pandemic amongst university older employees (age 50+). Participants (n = 846) were recruited throughout campus and completed an online survey. Summary statistics were used to describe characteristics of the study participants and frequency and level of UDWC and RSQ. Proportional odds models were used to assess major factors associated with UDWC and RSQ. The results showed 416 (43.2%) participants reported UDWC and 474 (49.2%) RSQ. Age was inversely, and obesity positively associated with UDWC and RSQ. With each 5-year increase in age, the OR (95% CI) was 0.87 (0.78, 0.97) for reporting UDWC and 0.90 (0.81, 1.00) for reporting RSQ. Obese individuals were significantly more likely to report a worse UDWC and RSQ (OR (95% CI) = 1.58 (1.18, 2.11) and 1.56 (1.16, 2.10) respectively).

